# The current role of surgery for single brain metastases

**DOI:** 10.1016/j.bas.2025.105600

**Published:** 2025-09-06

**Authors:** Tunc Faik Ersoy, Daniel Brainman, Diyan Dimov, Roland Coras, Björn Berger, Florian Weissinger, Matthias Simon

**Affiliations:** a Department of Neurosurgery, University Hospital OWL, Evangelisches Klinikum Bethel, Bielefeld, Germany; b Department of Neuropathology, University Hospital Erlangen, Friedrich-Alexander-University Erlangen-Nürnberg, Erlangen, Germany; c Department of Neuroradiology, University Hospital OWL, Evangelisches Klinikum Bethel, Bielefeld, Germany; d Department of Hematology, Oncology and Palliative Care, University Hospital OWL, Evangelisches Klinikum Bethel, Bielefeld, Germany

**Keywords:** Single brain metastasis, Residual tumor, Survival, Functional outcome, Progression-free survival, MRI

## Abstract

**Introduction:**

Surgery for singular brain metastases (BM) aims to improve survival by providing control of CNS disease. This concept may need to be refined against the background of recent advances in medical and radiation oncology. In particular there is a debate about the prognostic role of residual tumor.

**Research question:**

Does extent of resection influence overall- and cerebral progression-free-survival as well as functional outcome?

**Materials and methods:**

This retrospective series comprised 202 patients with single BM who underwent surgery 2015–2023. All patients underwent pre- and postoperative MR imaging.

**Results:**

Surgical indications included a tumor too large for radiosurgery (≥15.0 cm^3^; 42.5 %) and tissue acquisition for molecular analyses for potential therapeutic targets (21.8 %). Extent of resection was categorized as incomplete (8.4 %), questionably complete (22.3 %), and complete (69.3 %). Median residual tumor volume was only 0.12 cm^3^ (IQR 0.04–0.35). Complete resection was not correlated with better overall (OS) or CNS progression free survival. Complication rates and postoperative KPS did not vary significantly with resection category. Postoperative MRI revealed two cases with unilateral sigmoid sinus thrombosis in asymptomatic patients (cf. 11/16 [69.0 %] complications requiring treatment in symptomatic patients). We recorded 6.2 % major (CTCAE III-V) surgical, 5.3 % neurological, and 7.2 % medical complications. Postoperative treatment and major complications were prominent prognostic factors for OS.

**Conclusion:**

Small tumor remnants may have limited impact on survival. In the era of targeted therapies tissue acquisition for molecular analysis is an increasingly important indication for BM surgery.

## Highlights

• Residual tumor does not worsen overall survival or local disease control.
• Postoperative treatment is decisive for a prolonged survival.
• Surgery helps retain functional health to keep patients oncologically treatable.
• Extent of resection does not improve functional outcome.
• Postoperative MRI is presumably ubiquitous for asymptomatic patients.

## 1 Introduction

The principal role of surgery for single brain metastases (BM) has been established quite a while ago (Carapella et al., 2018; Ene and Ferguson, 2022; Patchell et al., 1990; Vecht et al., 1993). Surgery for single BM aims to control CNS disease, which in turn should lead to improved survival. This concept may need to be refined against the background of recent advances in medical and radiation oncology. Operations are often performed primarily in patients with large space-occupying tumors or tumors in (semi-)eloquent locations in order to prevent or improve neurological deterioration and keep patients capable of receiving further oncological therapy (e.g. tumors in the posterior fossa with the attendant risk of occlusive hydrocephalus and brainstem compression). Single fraction radiosurgery is an alternative for tumors < 3 cm (or < 15 cm^3^) (Soffietti et al., 2020; Le Rhun, 2021; Vogelbaum et al., 2021), however, the threat to neurological function posed by the extent of perifocal edema and tumor location nevertheless not rarely argues for resective surgery in such patients. Not uncommonly, surgery is required for tissue acquisition in cases without a known primary tumor or extracerebral metastases (Carapella et al., 2018; Ene and Ferguson, 2022; Ferguson et al., 2017). With the emergence of systemic therapy options, which are also effective against the cerebral disease (Le Rhun, 2021; Lin et al., 2023; Hurvitz et al., 2023), tissue acquisition for further molecular genetic diagnostics is playing an increasing role.

Extent of resection has been explored as a potential prognostic factor. The prognostic value of residual tumor remains controversial, with some studies reporting a poorer overall outcome and others not being able to confirm this (Lee et al., 2013; Jünger et al., 2021; Olesrud et al., 2019; Baumgart et al., 2024; Winther et al., 2022). More aggressive surgery likely leads to better local control (Winther et al., 2022; Kamp et al., 2015), but better control of CNS disease may not necessarily result in longer overall survival (Kocher et al., 2011; Mahajan et al., 2017). The potential benefits of surgical aggressiveness need to be balanced against surgery-related risks. We and others have shown that major complications often preclude postoperative treatment resulting in severely shortened survival (Ene and Ferguson, 2022; Ferguson et al., 2017; Ersoy et al., 2021, 2024).

This debate naturally involves the question of the role of early MR imaging following BM surgery in general. Recent studies demonstrated that surgeons often overestimate their resection rates (Olesrud et al., 2019; Kamp et al., 2015). Postoperative MRI scans can reveal complications such as ischemic lesions, bleedings, and venous sinus thrombosis (VST) which may correlate with postoperative neurological deterioration and require treatment (Gempt et al., 2013).

In this study, we report our institutional experience with surgical treatment for single BM in a large and recent patient cohort with early postoperative MR imaging. We specifically investigated the extent of resection and its potential impact on survival and functional outcomes.

## 2 Materials and Methods

### 2.1 Patients & surgery

We identified all patients with a single BM who underwent a craniotomy and tumor resection in our department between January 2015 and December 2023. Patients without postoperative MRI imaging, recurrent (after surgery or radiotherapy/radiosurgery) or leptomeningeal disease were excluded, leaving 202 consecutive patients (209 surgeries, i.e. seven patients underwent a second surgery for the removal of residual tumor) for further analysis.

All patients were discussed in our interdisciplinary neuro-oncological tumor board – and if necessary, in disease-specific tumor boards. We routinely aimed at a complete resection whenever safely feasible. Neuronavigation, intraoperative neuromonitoring and awake craniotomies were routinely used at the discretion of the operating surgeon (Sawaya et al., 1998).

### 2.2 Clinical data and follow-up

We reviewed the patients’ charts and all medical, pathology, and radiology reports for pertinent clinical, surgical, and radiological data. If necessary structured telephone interviews were performed to collect follow-up information. Comorbidities were assessed as for the calculation of the modified frailty index-5 (Weaver et al., 2019) with the inclusion of renal function impairment. We utilized the CTCAE classification framework (Common Terminology Criteria for Adverse Events v5.0; https://ctep.cancer.gov) to assess complications in a standardized fashion. Complications were recorded in three categories: surgical, surgery-related medical, and new persisting (≥30 days following surgery) focal neurological deficits. Functional outcome was assessed by the postoperative Karnofsky-Performance-Score (KPS).

### 2.3 Imaging data and volumetric analysis

All patients had adequate pre- and postoperative MR imaging (DWI, T2, SWI, 3D T1 native, FLAIR fs, 3D T1 ce). The majority of the patients received an MRI scan within 24 h after surgery (90.4 %). Fourteen patients received their postoperative MRI scan within 48 h and four patients within 72 h. One patient received her scan 5 days after the surgery and one after 7 days. All imaging studies were reviewed by a board-certified neuroradiologist (BB) blinded to the respective case's clinical course. We recorded the extent of resection qualitatively as complete, questionably complete, and incomplete (Fig. 2). For statistical analysis, patients with questionably complete resection were analyzed together with those with an incomplete resection. Pre- and postoperative tumor volumes were quantitatively assessed using a commercially available software (iplanNet, Brainlab AG, Munich, Germany). MR studies were also evaluated for the occurrence of ischemic lesions (territorial artery, terminal branch or venous infarction, unclear) (Gempt et al., 2013), surgery-related intra- or extra-axial bleeding > 1 cm (Fukamachi et al., 1986), (pneumo-)hydrocephalus, and venous sinus thrombosis.

**Fig. 1:**
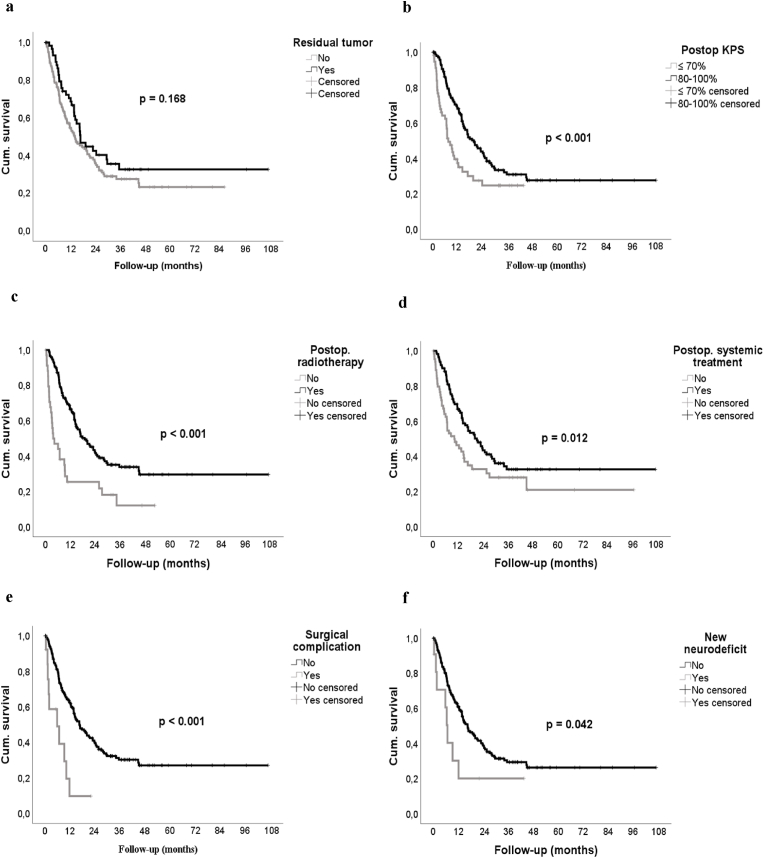
Univariate prognosis parameters for overall survival (Kaplan-Meier-estimates). **a** Extent of resection: Residual tumor had no statistically significant impact on overall survival. **b** Postoperative KPS: Superior overall survival was seen in patients with a good postoperative functional health status. **c** Postoperative radiotherapy & **d** postoperative systemic therapy: Patients who were not able to receive postoperative treatment had very poor survival outcomes. **e** Major surgical complications & **f** new major neurological deficit: Major complications worsen survival very significantly.

**Fig. 2:**
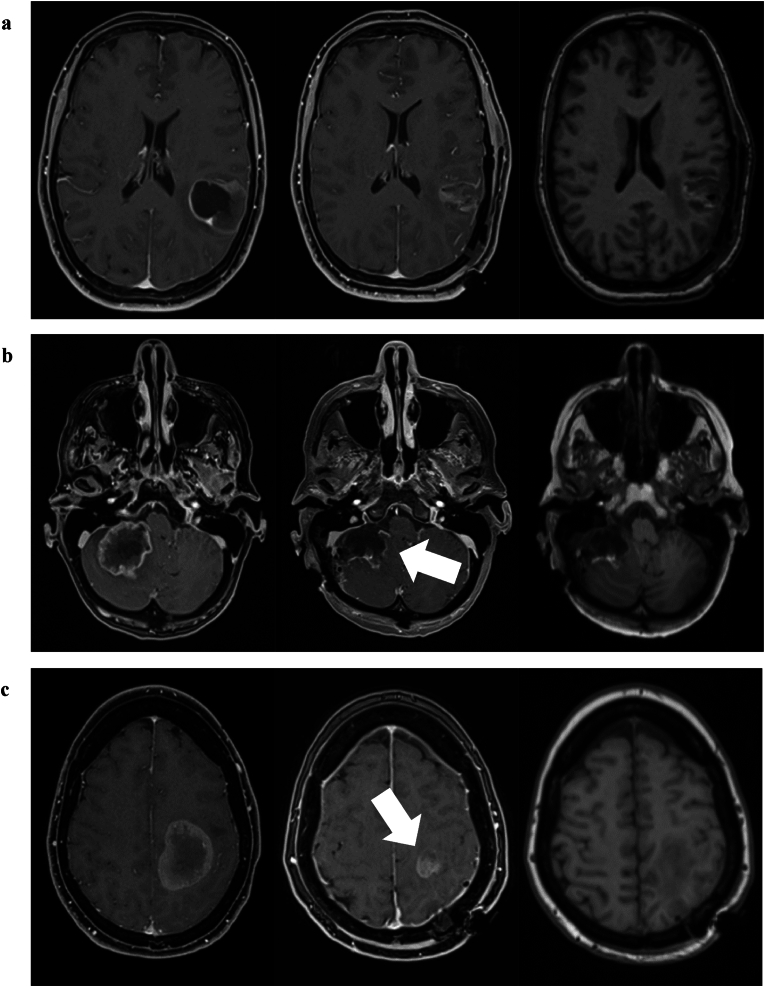
Extent of resection assessed by early postoperative MRI. **a** 46-year-old female with HER2 positive, ER/PR negative breast cancer. She suffered a secondary generalized focal onset seizure which led to cranial imaging and the diagnosis of a single 40 mm brain metastasis. Surgery was uneventful. No postoperative contrast enhancement, i.e. complete resection. **b** 57-year-old female with non-small cell lung cancer. She presented with headaches, dizziness, nausea and emesis in a short period of time. Surgery was performed to remove the space-occupying lesion in the posterior fossa which compressed the IV. ventricle and carried a risk for occlusive hydrocephalus. Very thin shell-like layer of contrast enhancement on the medial resection border (white arrow). The neuroradiological review classified this as questionable residual tumor. **c** 54-years-old female with an adernocarcinoma of the pancreas. The patient had developed mild hemiparesis on the right side. Surgery was performed for cytoreduction but also aiming to prevent further neurological worsening. The patient indeed remained neurologically stable following the surgery. Nodular tumor remnant (white arrow) in the central region left behind during surgery because of (reversible) deterioration of intraoperative MEP recordings.

### 2.4 Statistical analysis

Statistical analyses were carried out with commercially available IBM SPSS Statistics for Windows software (Version 25.0, IBM Corp., Armonk, NY). Categorical data was analyzed using Chi-squared-test and Fischer's exact test. OS and PFS was studied using Kaplan-Meier estimates. (Cox) regression analyses were employed for multivariate survival analyses. The alpha value for all tests was set to p < 0.05.

## 3 Results

### 3.1 Patient cohort and surgical treatment

The series comprised 102 (50.5 %) females and 100 (49.5 %) males. Median age was 64.0 (IQR 25–75 %: 57.0–72.0) years. Median pre- and postsurgical KPS were 90 % (IQR 25–75 %: 70–90 % and 70–92.5 %, respectively). The most frequent histology was lung cancer (94/202 = 46.5 %), followed by breast cancer (30/202 = 14.9 %) and malignant melanoma (15/202 = 7.4 %).

Median preoperative tumor volume was 12.25 (5.64–25.28) cm^3^ and median residual tumor volume (excluding cases with complete resections) was 0.12 (IQR 25–75 %: 0.04–0.35; all cases, median: 0, IQR 25–75 %: 0–0.03) cm^3^. We recorded complete resections in 140 patients (69.3 %; including 7 cases with two surgeries), questionably complete surgeries in 45 (22.3 %) and incomplete resections in 17 cases (8.4 %). 86 patients (42.6 %) had tumors ≥15.0 cm^3^ (believed to be too large for single fraction radiosurgery) and only 34 patients (16.8 %) harbored tumors ≤2 cm/4.0 cm^3 25^. Patients with an incomplete/questionably complete resection received postoperative radiotherapy more frequently (92.6 %; cf. complete resection 76.2 %, p = 0.012). Both groups did not differ with respect to the rate of systemic treatment (64.7 % vs. 62.4 %, p = 0.774).

In 44 (21.8 %) patients surgery was performed (at least in part) in order to obtain tissue for molecular studies. This includes 29 patients with breast cancer (14 tested positive for HER2), 13 with malignant melanoma (5 tested positive for the BRAF-V600E mutation), 1 with NSLSC (no PD-L-1 mutation), and 1 with esophagus cancer (no PD-L-1 mutation).

We observed 6.4 % (13/202 patients) major (CTCAE grades 3–5) surgical and 7.4 % (15/202) surgery-related medical complications as well as 5.2 % (11/202) new CTCAE grades 3–5 focal neurological deficits persisting at discharge. Further cohort characteristics can be found in Table 1, Table 2.

**Table 1:** Univariate prognosis parameters for functional outcome.

		Discharge KPS 80–100 %	p-value
**Age**	≥64.0 years (median)	61/101 (60.4 %)	<0.001
	<64.0 years	83/101 (82.2 %)	
**Sex**	Females	72/102 (70.6 %)	0.825
	Males	72/100 (72.0 %)	
**Preoperative KPS**^a^	80–100 %	133/150 (88.7 %)	<0.001
	≤70 %	11/52 (21.2 %)	
**Histology**	Lung	70/94 (74.4 %)	0.170
	Breast	24/30 (80.6 %)	
	Other	50/78 (64.1 %)	
**Presentation of disease**	Synchronous	48/66 (70.6 %)	0.753
	Metachronous	96/136 (66.4 %)	
**Location**	Supratentorial	113/145 (77.9 %)	<0.001
	Infratentorial	31/57 (54.4 %)	
**Comorbidities**	Yes	75/122 (61.5 %)	<0.001
	No	68/79 (86.1 %)	
**Extracerebral metastases**^b^	Yes	60/93 (64.5 %)	0.076
	No	70/91 (77.0 %)	
**Perioperative GPA**^c^	1	5/23 (21.7 %)	<0.001
	2	63/92 (68.5 %)	
	3 & 4	62/68 (91.2 %)	
**Preoperative seizures**	Yes	20/25 (80.0 %)	0.304
	No	124/177 (70.0 %)	
**Postoperative seizures (EPS**^d^**)**	Yes	3/6 (50.0 %)	0.242
	No	141/196 (71.9 %)	
**Preoperative tumor volume**	≥12.25 cm^c^ (median)	75/101 (74.3 %)	0.351
	<12.25 cm^c^	69/101 (68.3 %)	
**Extent of resection**	Complete	106/149 (71.1 %)	0.485
	Incomplete/questionably complete	52/66 (78.8 %)	
**Postoperative tumor volume**	≥0.12 cm^c^ (median)	27/32 (84.4 %)	0.482
	<0.12 cm^c^	24/31 (77.4 %)	
**Major surgical complication**	Yes	1/13 (7.7 %)	<0.001
	No	144/191 (75.4 %)	
**Major neurological complication**	Yes	0/11 (0.0 %)	<0.001
	No	143/189 (75.7 %)	
**Major medical complication**	Yes	5/15 (33.3 %)	<0.001
	No	139/187 (74.3 %)	

a KPS – Karnofsky Performance Status.

b Information missing for 18 patients.

c GPA – Graded Prognostic Assessment, information missing for 18 patients.

d EPS – Early postoperative seizures.

**Table 2:** Univariate survival prognosis parameters.

		N	mOS (95 %CI) in months	p-value
**Age**	≥64.0 years (median)	101 (50.0 %)	13.8 (9.0–18.6)	0.082
	<64.0 years	101 (50.0 %)	16.7 (11.7–21.8)	
**Sex**	Females	102 (50.5 %)	20.0 (14.0–26.0)	0.035
	Males	100 (49.5 %)	12.7 (8.4–17.1)	
**Preoperative KPS**^a^	80–100 %	149 (73.8 %)	17.1 (12.1–22.1)	0.109
	≤70 %	52 (26.2)	9.9 (3.5–16.4)	
**Postoperative KPS**	80–100 %	143 (70.8 %)	20.0 (15.0–25.0)	<0.001
	≤70 %	57 (29.2 %)	7.5 (4.6–10.4)	
**Histology**	Lung	94 (46.5 %)	14.0 (10.3–17.7)	0.322
	Breast	30 (14.9 %)	24.8 (12.2–37.5)	
	Other	78 (38.6)	16.8 (11.4–22.1)	
**Presentation of disease**	Synchronous	66 (32.7 %)	19.6 (12.2–27.0)	0.536
	Metachronous	136 (67.3 %)	14.6 (12.1–17.0)	
**Location**	Supratentorial	146 (72.7 %)	16.8 (11.8–21.7)	0.237
	Infratentorial	56 (27.3 %)	14.4 (9.0–19.7)	
**Comorbidities**	Yes	122 (60.0 %)	14.8 (10.8–18.9)	0.219
	No	80 (40.0 %)	17.1 (7.3–26.9)	
**Extracerebral metastases**^b^	Yes	93 (45.4 %)	14.6 (9.9–19.2)	0.159
	No	109 (54.6 %)	15.3 (6.2–24.4)	
**Perioperative GPA**^c^	1	23 (11.4 %)	6.6 (0.6–12.6)	0.004
	2	93 (46.0 %)	17.0 (12.1–21.8)	
	3 & 4	68 (24.7 %)	17.1 (5.7–28.6)	
**Preoperative seizures**	Yes	25 (12.4 %)	19.0 (7.4–30.6)	0.683
	No	177 (87.6 %)	15.3 (12.9–17.6)	
**Postoperative seizures (EPS**^d^**)**	Yes	6 (2.9 %)	6.9 (3.9–9.9)	0.287
	No	196 (97.1 %)	16.7 (12.9–20.5)	
**Preoperative tumor volume**	≥12.25 cm^c^ (median)	101 (50.0 %)	14.0 (9.9–18.1)	0.011
	<12.25 cm^c^	101 (50.0 %)	20.4 (13.3–27.6	
**Extent of resection**	Complete	139 (68.8 %)	14.4 (9.6–19.2)	0.168
	Incomplete/questionably complete	63 (31.2 %)	17.1 (12.3–22.0)	
**Postoperative tumor volume**	≥0.12 cm^c^ (median)	32 (50.8 %)	17.1 (12.3–21.1)	0.490
	<0.12 cm^c^	31 (49.2 %)	16.8 (9.3–24.3)	
**Postoperative radiotherapy**^e^	Yes	149 (73.8 %)	19.0 (13.2–24.8)	<0.001
	No	36 (26.2 %)	3.7 (0.0–7.8)	
**Postoperative systemic therapy**^f^	Yes	111 (55.0 %)	21.4 (15.5–27.4)	0.012
	No	67 (45.0 %)	10.4 (4.3–16.5)	
**Major surgical complication**	Yes	13 (6.2 %)	5.9 (0.0–13.2)	<0.001
	No	189 (93.8 %)	16.8 (12.6–21.0)	
**Major neurological complication**	Yes	11 (5.3 %)	6.9 (5.4–8.4)	0.042
	No	191 (94.7 %)	16.8 (12.8–20.7)	
**Major medical complication**	Yes	15 (7.2 %)	25.2 (0.0–50.9)	0.959
	No	187 (92.8 %)	16.2 (12.4–18.2)	

a KPS – Karnofsky Performance Status.

b Information missing for 18 patients.

c GPA – Graded Prognostic Assessment, information missing for 18 patients.

d EPS – Early postoperative seizures.

e Information missing for 17 patients.

f Information missing for 24 patients.

### 3.2 Functional outcome

**Fig. 3:**
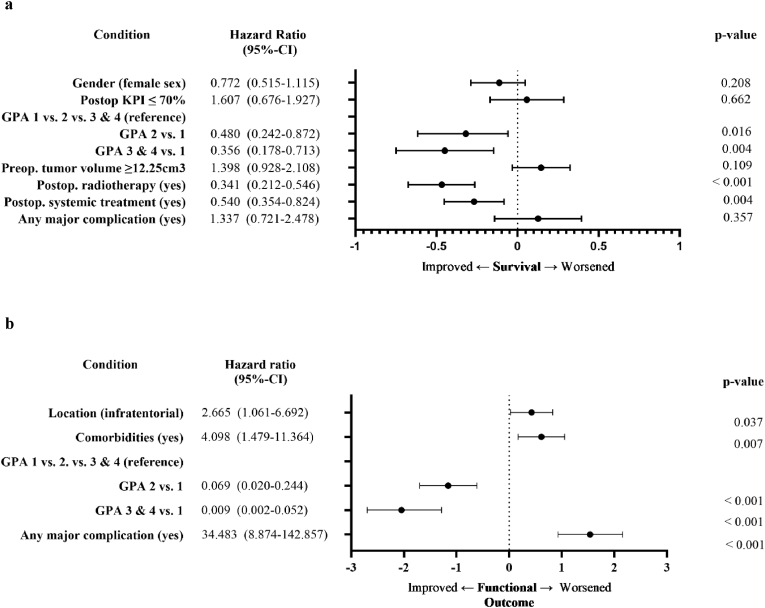
Multivariate cox regression and binary logistic regression analysis. **a** Cox regression analysis of univariate significant prognostic parameters for overall survival. **b** Binary logistic regression analysis of univariate prognostic parameters for functional outcome (i.e. postoperative KPS ≤70 % vs. 80–100 %). For statistical purposes, major complications were assessed together as “any major complication”. Age and preoperative KPS are part of the GPA score and were therefore not investigated separately even though both were significant univariate outcome predictors.

The majority of the patients retained their preoperative KPS following surgery (117/202 [57.9 %]). Fifty-one patients (25.2 %) had an improvement in their KPS with 17 of them showing an improvement of at least 20 %, while 34 patients worsened (16.8 %; ≥20 %: 20/202 [9.9 %]). Extent of resection was not statistically associated with functional outcome (postop. KPS 80–100 % and complete vs. questionably complete vs. incomplete/resection: 94/140 [67.1 %] vs. 37/45 [82.2 %] vs. 13/17 [76.5 %], p = NS). Extent of resection was also not associated with the occurrence of surgical, neurological or medical complications. Univariate predictors of a favorable postoperative functional outcome (i.e. postoperative KPS 80–100 %) were younger age, a good preoperative functional health status, lack of comorbidities, supratentorial disease, no complications, and a higher perioperative GPA score (Table 1). Functional health status, no complications, and GPA score retained significance in the multivariate analysis (Fig. 3b).

Patients with a poor postoperative functional outcome (i.e. KPS ≤70 %) were much less likely to receive postoperative treatment (radiotherapy, KPS ≤70 % vs. 80–100 %: 33/147 [22.4 %] vs. 114/147 [77.6 %], p = 0.001; systemic treatment, KPS ≤70 % vs. KPS 80–100 %: 26/110 [24.6 %] 84/110 [76.4 %], p = 0.082). Major surgical complications often precluded postoperative treatment (radiotherapy: 5/11 [45.5 %] cf. without complications: 144/173 [83.3 %], p = 0.002; systemic treatment: 4/11 [36.4 %] cf. without complication 107/165 [65.0 %], p = 0.058). Patients with new persisting focal neurological deficits were less likely to receive postoperative radiotherapy (5/10 [50.0 %], cf. without complication: 144/173 [83.3 %], p = 0.010), whereas those with medical complications were less likely to receive postoperative systemic treatment (6/15 [40.0 %], cf. without: 105/161 [65.2 %], p = 0.053).

### 3.3 Survival analyses

Median overall survival (mOS) was 15.7 months (95 % CI: 12.4–19.0). One hundred thirty-six patients (67.3 %) had already died at the time of analysis. Cause of death could be ascertained in 79 patients (58.1 %). 46 patients (58.2 %) died from complications of their systemic disease or other non-CNS-disease-related causes.

Extent of resection did not influence OS (mOS complete resection: 14.4 months [9.6–19.2] vs. incomplete/questionably complete resection: 17.1 months [12.3–22.0], p = 0.168). Statistically significant survival predictors included female sex, a higher postoperative KPS (>70 %), postoperative radio- and systemic treatment, as well as no major surgical complications or new neurodeficits (Table 2 and Fig. 1). Patients with smaller tumors had better survival outcomes (≥12.3 cm^3^: 14.0 months [9.9–18.1] vs. < 12.3 cm^3^: 20.4 months [13.3–27.6], p = 0.011). GPA score and postoperative radiotherapy/systemic treatment remained significant survival predictors in the multivariate Cox analysis (Fig. 3a).

Only limited information on CNS progression was available (after exclusion of patients who died within 30 days of surgery: 142 patients [70.3 %]). Fifty-six patients (27.6 %) developed recurrent CNS disease. Extent of resection and postoperative radiotherapy did not significantly influence CNS-PFS (1 yr. CNS disease control; complete vs. questionably complete/incomplete resection: 49.6 % vs. 61.9 %, p = 0.190 & RT vs. no: 54.3 % vs. 53.9 %, p = 0.468).

### 3.4 Postoperative neuroimaging: complication detection and management

Early postoperative imaging revealed complicative findings in 113 patients including 83 ischemic lesions (terminal branch: 34, territorial branch: 12, venous associated: 34, unclear: 6), 20 intra-axial and 8 extra-axial hemorrhages >1 cm, 7 venous sinus thromboses (all unilateral transverse/sigmoid sinus; tumor location - posterior fossa: 6, temporo-basal: 1), and (pneumo-)hydrocephalus in 11 cases. Only 16 patients (9.7 %) showed clinical symptoms which required surgical interventions in 8 cases (EVD: 1, craniotomy: 3, both: 4). Except for 2 patients with clinically silent unilateral sigmoid sinus thrombosis, none of the asymptomatic patients received specific treatment.

## 4 Discussion

For the present paper we have analyzed our institutional experience with the surgical management of patients with single brain metastases. Median OS was 15.7 months in our cohort which is in line or even compares favorably with the more current literature (Jünger et al., 2021; Olesrud et al., 2019; Baumgart et al., 2024; Winther et al., 2022). It appears that survival in this patient group has improved considerably over the last decades (Patchell et al., 1990).

We specifically studied the potential impact of residual tumor on survival and did not observe a survival benefit derived from complete resections in our series. Several studies have investigated this issue and conflicting results have been published. The rate of complete resections in our cohort was 68.8 %, which is comparable to the figures reported by others (61.5–72.6 %) (Jünger et al., 2021; Olesrud et al., 2019; Baumgart et al., 2024; Winther et al., 2022). However, published series might differ with respect to residual tumor volumes. Median residual tumor volume in our series (including those patients with a questionably complete resection was merely 0.12 cm^3^ (overall: 0 cm^3^). Of note, in the cohort reported by Olesrud et al. (2019), 22 % of the patients had measurable residual tumor > 1 cm (in one dimension) and these authors observed a correlation between extent of resection and survival. Only 12 cases (6.1 %) in the series by Jünger and co-workers (Jünger et al., 2021) (who found no prognostic impact of residual tumor on survival) harbored >5 % residual tumor (cf. 2 cases [1.0 %] in the present cohort). In conclusion our data may not necessarily argue against the value of complete resections for brain metastases but rather show that very small residual tumor volumes have no major prognostic impact.

Another reason why a survival benefit derived from a complete resection of brain metastases is not seen in all studies might be that cohorts differ with respect to the intensity of adjuvant therapies. Except for Jünger et al. (2021), studies have not investigated the effect of postoperative systemic therapy on overall survival. Our data confirms postoperative systemic therapy as a major prognostic factor. Olesrud et al. (2019) and Winther et al. (2022) both report that complete resections have a positive survival impact, and 58.0 % and 61.0 % radiotherapy rates, respectively. For comparison, in the present and in the series by Jünger et al. and Baumgardt and co-workers (Jünger et al., 2021; Baumgart et al., 2024) - which found no correlation between extent of resection and survival - 73.5–100 % of cases had some form of postoperative radiation therapy. Radiotherapy was associated with improved survival in this and in the cohort by Baumgardt et al. (Baumgart et al., 2024).

However, the impact of CNS radiotherapy on survival is likely complex. Randomized trials investigating the role of radiotherapy and stereotactic radiosurgery after surgery for brain metastases have shown that these adjuvant treatments lower the in-brain recurrence rate but not necessarily improve overall survival (Kocher et al., 2011; Mahajan et al., 2017). Likely due to the retrospective nature of most studies and non-standardized (imaging) follow-up not much data on CNS PFS after brain metastases surgery is available. Similar to Jünger et al. (2021), we observed no correlation between residual tumor and CNS recurrences. Kamp et al. (2015) reported a higher in-brain recurrence rate in patients with residual tumor, however, no such association with recurrence of the index tumor.

The most prominent cause of conflicting data might well be the heterogeneity of the disease and therefore of the various patient cohorts under investigation. E.g. in contrast to the series by Baumgardt et al. the presence of extracerebral metastases was not prognostic in our cohort. Some histologies are considered radio-resistant and there is no reason to believe why different histologies should not respond differently also to surgery. Survival indeed varied in this and in many published cohorts with histology (Baumgart et al., 2024; Winther et al., 2022). Neurosurgeons should probably distance themselves from perceiving BM as a single entity, and distinguish between cases according to histology (and possibly in the near future molecular findings as well). Of note, in about 20 % of our patients, surgery was indicated at least in part to allow for tissue acquisition for molecular genetic analysis. This figure may serve to illustrate the increasingly important role of diagnostic surgery for BM in the era of targeted therapies (Le Rhun, 2021).

Functional outcomes were very reasonable, and complication rates (major persisting new neurological deficits: 5.4 %, surgical/medical complications: 6.4/7.4 %) were relevant but generally quite acceptable, and in line with the corresponding figures in the literature (Jünger et al., 2021; Winther et al., 2022). This is important since many (42.6 %) of our patients had large space-occupying tumors (≥15 cm^3^) not eligible for single fraction radiosurgery, and only 16.8 % of our cases had tumors <2 cm (Le Rhun, 2021; Shaw et al., 2000). Most patients were able to retain or improve their functional health through surgery, which in turn is a prerequisite for further postoperative treatment and therefore a very strong survival predictor. The potential role of surgery in maintaining or even restoring (neurological) function should not be underestimated.

Routine postoperative MR imaging not only allows to assess residual tumor, but also certain surgery-related complications. Indeed, pathological MRI findings were noted in more than half of our cases. However, only 9.7 % of the patients were symptomatic and except for two patients with a clinically silent unilateral sigmoid sinus thrombosis who were put on prophylactic anticoagulant therapy for 6 weeks none of the asymptomatic patients received treatment (cf. 8/16 [50.0 %] surgical interventions in symptomatic patients). These figures do not necessarily support the current standard of obtaining routine postoperative MRIs in patients undergoing brain metastases surgery (Le Rhun, 2021). We will nevertheless continue with this practice since postoperative MR imaging provides the surgeon with feedback with respect to appropriate tissue handling (Gempt et al., 2013) and the quality of the resection. Previous studies (Olesrud et al., 2019; Kamp et al., 2015) have shown that the surgeon's intraoperative impression and the actual completeness of a resection quite often differ considerably.

Finally, our study has significant limitations including its retrospective design, the heterogeneity of the patient cohort and selection bias, i.e. only preselected patients with cerebral metastases will be considered for surgery by their treating oncologists. Postoperative medical and radio-oncological information was not available for all patients, and follow-up was not standardized. On the other hand, we were able to study a fairly large and very recent patient cohort. We investigated the role of postoperative systemic therapy and complication assessment was performed in a standardized fashion using a well-established classification.

## 5 Conclusion

Small tumor remnants following surgery for single brain metastases may have a limited impact on survival, in particular in patients undergoing postoperative radiation and systemic treatment. Surgery often preserves or even improves neurological function. In the era of targeted therapies tissue acquisition for molecular analyses is already a prominent indication for surgery. Routine postoperative MR imaging is of limited value with respect to surgical complication management.

## Consent to participate and publish

Due to the retrospective nature of the study, the responsible institutional research committee (Ethikkommission der Ärtzekammer Westfalen-Lippe) and local law waived the need of obtaining informed consent. No personal data is published in this manuscript; thus this is not applicable for this study.

## Author contributions

Conceptualization: [TFE, MS]; Methodology: [TFE, MS], Formal investigation and analysis: [TFE, DB, DD, BB]; Writing - original draft preparation: [TFE]; Writing - review and editing: [TFE, BB, RC, FW, MS]; Figures and Tables [TFE] Resources: [BB, RC]; Supervision: [MS]. All authors read and approved the final manuscript.

## Ethics & inclusion statement

This study was performed in line with the principles of Declaration of Helsinki. Approval was granted by the Ethics Committee of Ärtzekammer Westfalen-Lippe und der Westfälischen Wilhelms-Universität Münster, Germany (Az, 2021-073-f-S)

## Funding

The authors declare that no funds, grants, or other support were received during the preparation of this manuscript.

## Declaration of competing interest

The authors declare that they have no known competing financial interests or personal relationships that could have appeared to influence the work reported in this paper.

## Data Availability

The datasets generated during and/or analyzed during the current study are available from the corresponding author on reasonable request.

## References

[bib1] Baumgart L. (2024). Single brain metastases – prognostic factors and impact of residual tumor burden on overall survival. Front. Oncol..

[bib2] Carapella C.M., Gorgoglione N., Oppido P.A. (2018). The role of surgical resection in patients with brain metastases. Curr. Opin. Oncol..

[bib3] Ene C.I., Ferguson S.D. (2022). Surgical management of brain metastasis: challenges and nuances. Front. Oncol..

[bib4] Ersoy T.F. (2021). Surgical treatment of cerebellar metastases: survival benefits, complications and timing issues. Cancers (Basel).

[bib5] Ersoy T.F. (2024). Defining the role of surgery for patients with multiple brain metastases. J. Neuro Oncol..

[bib6] Ferguson S.D. (2017). Neurosurgical management of brain metastases. Clin. Exp. Metastasis.

[bib7] Fukamachi A., Koizumi H., Nagaseki Y., Nukui H. (1986). Postoperative extradural hematomas: computed tomographic survey of 1105 intracranial operations. Neurosurgery.

[bib8] Gempt J. (2013). Postoperative ischemic changes following brain metastasis resection as measured by diffusion-weighted magnetic resonance imaging: clinical article. J. Neurosurg..

[bib9] Hurvitz S.A. (2023). Trastuzumab deruxtecan versus trastuzumab emtansine in patients with HER2-positive metastatic breast cancer: updated results from DESTINY-Breast03, a randomised, open-label, phase 3 trial. Lancet.

[bib10] Jünger S.T. (2021). The debatable benefit of gross-total resection of brain metastases in a comprehensive treatment setting. Cancers (Basel).

[bib11] Kamp M.A. (2015). Early postoperative magnet resonance tomography after resection of cerebral metastases. Acta Neurochir (Wien).

[bib12] Kocher M. (2011). Adjuvant whole-brain radiotherapy versus observation after radiosurgery or surgical resection of one to three cerebral metastases: results of the EORTC 22952-26001 study. J. Clin. Oncol..

[bib13] Le Rhun E. (2021). EANO-ESMO clinical practice guidelines for diagnosis, treatment and follow-up of patients with brain metastasis from solid tumours. Ann. Oncol..

[bib14] Lee C.H. (2013). The role of surgical resection in the management of brain metastasis: a 17-year longitudinal study. Acta Neurochir (Wien).

[bib15] Lin N.U. (2023). Tucatinib vs placebo, both in combination with trastuzumab and capecitabine, for previously treated ERBB2 (HER2)-positive metastatic breast cancer in patients with brain metastases: updated exploratory analysis of the HER2CLIMB randomized clinical trial. JAMA Oncol..

[bib16] Mahajan A. (2017). Post-operative stereotactic radiosurgery versus observation for completely resected brain metastases: a single-centre, randomised, controlled, phase 3 trial. Lancet Oncol..

[bib17] Olesrud I.C. (2019). Early postoperative MRI after resection of brain metastases—complete tumour resection associated with prolonged survival. Acta Neurochir (Wien).

[bib18] Patchell R.A. (1990). A randomized trial of surgery in the treatment of single metastases to the brain. N. Engl. J. Med..

[bib19] Sawaya R. (1998). Neurosurgical outcomes in a modern series of 400 craniotomies for treatment of parenchymal tumors. Neurosurgery.

[bib20] Shaw E. (2000). Single dose radiosurgical treatment of recurrent previously irradiated primary brain tumors and brain metastases: final report of RTOG protocol 90-05. Int. J. Radiat. Oncol. Biol. Phys..

[bib21] Soffietti R., Ahluwalia M., Lin N., Rudà R. (2020). Management of brain metastases according to molecular subtypes. Nat. Rev. Neurol..

[bib22] Vecht C.J. (1993). Treatment of single brain metastasis: radiotherapy alone or combined with neurosurgery?. Ann. Neurol..

[bib23] Vogelbaum M.A. (2021). Treatment for brain metastases: ASCO-SNO-ASTRO guideline. J. Clin. Oncol..

[bib24] Weaver D.J. (2019). The modified 5-Item frailty index: a concise and useful tool for assessing the impact of frailty on postoperative morbidity following elective posterior lumbar fusions. World Neurosurg..

[bib25] Winther R.R. (2022). Surgery for brain metastases—impact of the extent of resection. Acta Neurochir (Wien).

